# Consistency in decision-making between survey teams and the decision-making body in a professional education program accrediting agency

**DOI:** 10.15694/mep.2019.000113.1

**Published:** 2019-05-28

**Authors:** Robert Hash

**Affiliations:** 1 American Medical Association

**Keywords:** accreditation, peer review, consistency, educational program

## Abstract

This article was migrated. The article was marked as recommended.

Credibility of an education program accreditor is dependent on consistency in decision-making among program reviews. The use of peer-review accreditation survey teams is a potential source of inconsistency in the review of individual programs, especially so when the agency employs non-prescriptive accreditation standards. The accrediting agency that is the subject of this study utilized multiple steps to ensure consistency in the recommendations from peer survey teams. Analysis of the agreement between survey team recommendations and final decisions by the decision-making body revealed a coefficient of agreement of 0.927, indicated a high degree of agreement. The results suggest the accrediting agency’s processes are effective in ensuring that peer review survey teams are applying the accreditation standards consistently between peer teams and the accrediting agency.

## Introduction

An accrediting agency’s credibility is judged, in part, by consistency in decision-making. To this point, the United States Department of Education’s regulations for recognized accrediting agencies stipulates in regulation 602.18(b) that the agency “has effective controls against the inconsistent application of the agency’s standards.” ( United States Department of Education, 2012)As noted by Hunt et al, “Standards are the lens through which accreditation views the world, creating the framework for consistency in review across programs ( Hunt *et al*., 2016).

A key factor in reliable and accepted educational program accreditation is that accreditation standards are judged in a consistent manner within and across institutions over time ( Barzansky, Hunt, and Busin, 2013). A criticism of accreditors has been a perception of inconsistency in how agency standards are applied to individual programs ( Greenfield *et al*., 2013). For reasons that will be discussed, a perceived and potential source of inconsistency in the application of accreditation standards - particularly non-prescriptive standards - occurs at the level of the survey teams.

Despite the importance of consistency in application of standards between programs, little work has been published on processes used to achieve consistency, and the effectiveness of these processes. This manuscript will describe the steps a programmatic accreditor has taken toward achieving consistency in decision-making between survey teams and the decision-making body, and provide outcomes data on the effectiveness of the process.

### Description of the agency’s accreditation process

Although the study agency uses the term “Elements” for the level of survey team review, the Elements serve the same role as “standards” for many accreditors. “Elements” will be used in the description of the agency’s processes and outcomes measures, and “Standards” will be used as a comparable term elsewhere.

The accreditation agency employs 93 Elements formatted as declarative statements of expectations for the educational programs ( Liaison Committee on Medical Education, 2016). All but two of the Elements are non-quantitative, with no formal performance benchmarks, and most are non-prescriptive, that is, they allow expectations to be met in different ways across schools without expectations for specific numbers or policies. Two of the Elements only apply to programs with regional campuses.

The term “accreditation” as used here is for the periodic accreditation site surveys and survey reports that are required for continuing accreditation of educational programs previously accredited by the agency.

The initial steps in a full accreditation survey are a self-study by the program and completion of a directed-inquiry data collection instrument (DCI) that includes questions related to each Element. The DCI is formatted to align with the structure of the team report. The survey team will review the DCI, perform an on-site survey, and make one of the following recommendations on performance regarding the program’s performance for each of the Elements: Satisfactory, Satisfactory with a need for Monitoring, or Unsatisfactory (see Appendix for definitions). The team report, which includes quantitative and qualitative information for each element, provides justification for that recommendation in the form of data or description of findings. The survey report therefore provides the basis for performance decisions by the decision-making body of the agency (the Committee) for each of the Elements. The Committee reviews the team’s recommendations and accompanying data and report, and either agrees with the survey team or changes the recommendation for the final determination for each Element.

### Steps that are used by the agency to promote consistency

Survey team member selection, survey team organization, standardization of reporting formats, survey team training, and agency support have been shown to be factors affecting consistency in decision-making for an accrediting agency ( Greenfield *et al*., 2009), ( Greenfield *et al*., 2015), ( Greenfield *et al*., 2016). With the largely non-prescriptive and non-quantitative nature of the agency’s Elements, consistency requires that survey teams and the Committee have similar expectations of what constitutes satisfactory performance in the Elements. Several processes are in place within the agency to support consistency, as noted in the following paragraphs.

*Team composition and training:* Team composition and team member duties are designed to provide consistency in on-site evaluation. Survey teams typically consist of five or six members, selected from a pool of more than 100 volunteer surveyors. Most teams include at least one member of the Committee, and two or three volunteer professional members with experience on survey teams, and an experienced team secretary. Inexperienced members receive additional supervision and mentoring by the team chair and team secretary. Team members are required to attend an annual team-training webinar, which includes discussions on how to evaluate a school’s performance for many of the elements. Team members are evaluated after the survey visit for their knowledge of accreditation expectations, preparation, writing ability, and contributions to the team.

Team secretaries are responsible for assembling and editing survey reports, and are either professional agency staff, Committee members, or professional educators who are contracted for this purpose after demonstrating exceptional team and writing skills in previous team assignments. They perform multiple surveys each year, receive additional training, and may attend Committee meetings. The team secretaries also meet annually to discuss the application of the elements and provide feedback to the agency on the visit process and the interpretation of elements. Through these steps, the team secretaries add to consistency through experience, interpretation of data, and providing the rationales for team recommendations for performance in elements. The presence of an accreditation Committee member and/or a professional staff member on the survey team is intended to improve the consistency and the credibility of the recommendations by providing team members with insight into how the Committee reviews the reports, views data, and interprets the accreditation elements. After accreditation action on a survey report, team members are sent a communication indicating the changes to team findings that were made by the Committee. This helps team members understand the expectations of the Committee and “calibrate” their responses in future surveys.

*Draft report review:* Before the Committee takes action on the final survey reports, draft versions of the reports are reviewed on multiple levels. The team secretaries compile and edit the team members’ writing assignments for accuracy, formatting, and completeness. The team secretaries’ drafts are then reviewed by two or more members of the agency’s professional staff for completeness, internal consistency, and to ensure that adequate documentation is included to support the team’s findings. Draft reports with comments are returned to the team secretary for review and editing as deemed appropriate by the team, and reviewed by leadership of the surveyed educational program for correction of factual errors.

*Committee structure:* The 17 professional and public Committee members serve staggered three-year terms, with the option for reappointment for a second term, which almost all members accept. This provides for significant “institutional memory” on the Committee. Each Committee member will review more than 100 survey reports and thousands of recommendations for elements during their tenures on the Committee.

*Informational publications:* There are numerous publications for schools, survey teams, and Committee members that aim at creating consistency in both format of reports, the process of visits, and interpretations of the intent of elements. These documents are available on the agency’s website. Both the DCI and the survey team report have standardized templates to ensure that teams and the Committee have a uniform set of information to inform the decision for each Element. For some Elements, the agency has produced guidance documents that explain the intent of specific accreditation elements and how the Committee applies Elements to programs. These documents, which are posted on the website serve to guide the schools, survey teams, reviewers, and Committee members and as they prepare and review reports.

## Methods

The author retrospectively examined survey teams’ recommendations and respective Committee determination of performance for 49 survey team reports for academic years 2015-16 through 2017-18. All full surveys during this period were included in the study. The author excluded the review of 18 Elements for each survey report, leaving 73 or 75 elements for review, depending on the structure of the program. The excluded Elements are either administrative in nature or historically were rarely, if ever, found by teams or the LCME to have performance concerns. This resulted in the analysis of 3665 Elements. The author identified and recorded each incident where there was discrepancy between survey team recommendations and final Committee decisions for an element.

## Results/Analysis

Of the 3665 recommendations on Elements, the Committee made a different recommendation on 136 (3.7%) of the Elements. The Committee agreed with the survey team recommendations for 96.3% of the Elements, resulting in a coefficient of determination (kappa statistic) of 0.927, indicating a high level of agreement. The kappa statistic was calculated to account for the possibility that reviewers may have had uncertainty about the correct assignment of performance ( McHugh, 2012).

Of the determination changes, the Committee assigned a more favorable determination of performance to 43 Elements, and a less favorable determination of performance for 93 Elements. Table 1 summarizes data on Committee changes to the Elements.

**Table 1.** Data on changes in Element performance determination

Total Elements recommendations by the survey team ^1^	3665
Number of survey team recommendations changed by the Committee	136
% of survey team recommendations changed by the Committee	3.7%
% of survey team findings confirmed by the Committee	96.3%
Coefficient of determination	.927
Number of more favorable determinations (% of total changes)	43 (32%)
Number of less favorable determinations (% of total changes)	93 (68%)

^1^ Elements that are administrative, or historically are never or rarely cited for performance concerns are not included

Of the total number of recommendations made by teams, the majority were for Satisfactory performance (3181, or 86.8%). Of the changes made to survey team recommendations by the Committee, more than half (56%) of the changes were made to “Satisfactory” performance recommendations. Data in Table 2 represents the number and direction (more severe or less severe) of changes to survey team recommendations made by the Committee for each category of Element performance.

**Table 2.** Number and “direction” of changes to survey team recommendations

Survey Team Recommendation	Total number (%) of changes	Changed to Satisfactory	Changed to Satisfactory with Monitoring	Changed to Unsatisfactory
Satisfactory	71 (56%)		63	8
Satisfactory with monitoring	42	20		22
Unsatisfactory	23	8	15	

## Discussion

Consistency in determinations of performance across individual programs is essential to the fair application of accreditation in higher education and the validity of accreditation in general ( Hinchcliff *et al*., 2016).

One potential source of inconsistency in the application of accreditation standards - particularly non-prescriptive standards - rests with the experience, bias, and knowledge of the surveyors. While non-quantitative standards allow for innovation and efficient resource allocation in disparate environments, this also creates the potential problem of determining a floor (minimal expectation) for satisfactory performance. Inconsistency in the decision of whether a program at least meets the floor is potentially amplified through the use of a pool of volunteer survey team members who may only participate in one survey visit per year. The author also acknowledges that comparing the agency’s decisions on each Element between accredited programs, while possibly a better measure of consistency, would be very difficult, as the agency’s standards and elements are intentionally non-prescriptive in nature for the purpose of allowing flexibility and innovation for the programs. The application of non-quantitative Elements often requires consideration of context, which means that programs may be meeting an Element in different ways or that the same strategy could be satisfactory for one program but not for another, which could lead to inconsistency in the review process.

Marre posited that an accrediting agency must ask itself three questions regarding consistency in decision-making: 1) are decisions based on criteria or standards, 2) is the agency interpreting the criteria or standards according to the intent of the criteria or standards, and 3) are the recommendations defensible over time (Marre 2014)? She notes that consistency in program accreditation “is not applying criteria or standards as a formula, or as imperatives, but about making decisions based on the intent or educational value of a criteria and applying it to the program being evaluated in context”. She noted that accreditation systems should have a multi-tiered structure to bring into consideration a variety of perspectives and guard against individual biases or dominance of an individual’s perspective. Eaton described the decision-making process regarding program quality as one of the four pillars of accreditation, along with scope of activity, standards and policies, processes, and decision-making judgements ( Eaton 2018). As described above, the study agency incorporated processes to ensure that multiple perspectives across multiple tiers were brought to bear in decision-making about performance in non-prescriptive standards.

Many programmatic accrediting agencies assemble survey teams of volunteers with educational and administration expertise in the profession and in the accreditation process for program review. This practice, while crucial to the ability of the accreditor to provide accreditation services at reasonable cost, is not without potential drawbacks. Each individual carries his/her own biases, experiences, and home institution perspectives into the process, with the potential for interpretations and recommendations to be affected by those biases. Experience in accreditation occurs by doing accreditation, which means that most surveyors must at some point be novices, enhancing the potential for variability in assessment of performance despite training activities provided by the agency. The team dynamic element also plays a role as survey teams reach consensus on program performance recommendations. The same potential drawbacks apply to the members of the Committee. These challenges can be mitigated through training of team and Committee members and by peer mentoring. For example, survey teams consist of experienced and novice members, as does the Committee.

Weaknesses of this study include assumptions that were made in the design of this study and interpretation of the data. The study assumed that the performance decision for each Element by the Committee was the “right” decision. While likely true most of the time, the Committee itself may have made the wrong decision for performance on some of the Elements. The study also assumed that the Committee reviewed each Element with a “Satisfactory” recommendation with the same attention to detail as recommendations for performance concerns. Experience with accreditation practices might suggest otherwise, thus introducing a potential bias in the data. The author attempted to mitigate some of this potential bias by excluding 18 of the 93 Elements rarely, if ever, not determined to be “Satisfactory”. The finding that the Committee changed more Satisfactory recommendations than performance concerns is reassuring that the Committee did indeed scrutinize Elements recommendations for Satisfactory performance.

## Conclusion

The author of this study used consistency of the outcome of the review process, from survey team recommendations to final decisions, as one determinate of the degree of consistency in the application of agency standards across programs between survey teams and the Committee.

In summary, the level of agreement between survey teams and the Committee was high regarding individual program performance on accreditation elements, supporting the notion that consistency can be achieved among survey teams in higher education accreditation.

To the author’s knowledge, this is the first publication that describes a higher education accreditation agency’s processes to achieve consistency in accreditation decisions between on-site survey teams and the agency review committee, and provides data on the effectiveness of those processes. Previous publications of a similar nature have used health care facility accreditation, rather than educational program accreditation. While there are similarities, it is not known if the principles fully apply and outcomes are generalizable across types of accreditation agencies.

## Take Home Messages

• Consistency in the application of accreditation standards across accredited programs is essential for a credible accreditation agency.
• Survey teams composed of volunteer peer reviewers, while adding value at low cost to accrediting agencies, are a potential source of inconsistency in the application of accreditation standards.
• A structured, multi-tiered accreditation report review process supports consistency in decision-making for accrediting agencies.
• Attention to survey team training and composition can lead to consistency in survey team recommendations about compliance with accreditation standards.
• Survey teams composed of peer volunteers can make recommendations for compliance with standards that are consistent across survey teams and between survey teams and the decision-making body.

## Notes On Contributors

Robert Hash, MD, MBA, currently serves as the LCME Assistant Secretary in the Chicago office of the Liaison Committee on Medical Education. His background includes private clinical practice, teaching and administration in medical schools, and participation on accreditation survey teams.

## Appendices

Glossary of terms

*Satisfactory:* The required policy, process, resource, or system is in place and, if required by the element, there is sufficient evidence to indicate that it is effective.

*Satisfactory with a need for monitoring:* The education program has the required policy, process, resource, or system in place, but there is insufficient evidence to indicate that it is effective. Therefore, monitoring is required to ensure that the desired outcome has been achieved; *or* the education program’s performance currently is satisfactory with respect to the element, but there are known circumstances that could directly result in unsatisfactory performance in the near future.

*Unsatisfactory:* The education program has not met one or more of the requirements of the element. The required policy, process, resource, or system either is not in place or is in place but has been found to be ineffective.
