# Supplementary material for: Consistency in decision-making between survey teams and the decision-making body in a professional education program accrediting agency
Source: MedEdPublish (2016). 2019 May 28;8:113. [Version 1] doi: 10.15694/mep.2019.000113.1 (PMC10712487; doi:10.15694/mep.2019.000113.1)
Supplement: Peer review history

# Reviewer report: Reviewer response for version 1

**DOI:** 10.21956/mep.19862.r30002

**Published:** 2020-05-13

**Authors:** Richard Hays

**Affiliations:** 1 James Cook University

This review has been migrated. The reviewer awarded 5 stars out of 5 Thanks to the author for being brave enough to open up a topic that could be controversial. The methods are clear and appropriate to the task. The description of how at least one accreditation agency (the LCME) approaches achievement of consistency is clear and welcome. It should also reassure team members and the faculty of of schools that are subject to accreditation processes, because the playing field should be level. As a participant in almost 30 accreditation teams in various jurisdictions, I can say that similar issues and processes are present in other major medical education accreditation systems. Great effort is exerted in training of assessors to understand measure against the criteria. Reports are moderated by more experienced assessors and by the committees that have the responsibility to make decisions that must be seen to defend patient safety and promote high quality of care. There is, of course, an alternate view. A heavily moderated system may be inherently conservative, being focused more on what 'is' and 'has been', and less able to make judgements about different or new ways that some institutions may like to try as they strive to meet standards. Therefore, new programs, major expansions and major curriculum revisions all require substantial consideration that may be better presented to accreditation agencies independently of the normal accreditation cycle. One aspect that I would have liked to see mentioned is the potential to use the list of 'most commonly disagreed' standards as worthy of further analysis to see if the standards and criteria require clarification or updating. This is a paper that should be read by all involved in accreditation.

Reviewer Expertise:

NA

No decision status is available

# Reviewer report: Reviewer response for version 1

**DOI:** 10.21956/mep.19862.r30000

**Published:** 2019-09-10

**Authors:** Ken Masters

**Affiliations:** 1 Sultan Qaboos University

This review has been migrated. The reviewer awarded 4 stars out of 5 A short but really interesting paper dealing with consistency in decision-making between survey teams and the decision-making body in a professional education program accrediting agency. As accreditation becomes ever more important, inconsistency in evaluation can be devastating to a department and a school, and the paper begins by clearly identifying the need for consistency in evaluation. The author follows with a knowledgeable and detailed account of the accreditation issues and processes involved. The Methods are succinct, but, perhaps a little short. It would have been useful if some more detail has been given about the institutions. There is a fair analysis of the results, and the authors has correctly noted the limitations, particularly that there has to be assumption of correctness. Overall, the consistency of the results are re-assuring to all institutions that have undergone accreditation, and those that plan to do so in the future. A well-written paper.

Reviewer Expertise:

NA

No decision status is available

# Reviewer report: Reviewer response for version 1

**DOI:** 10.21956/mep.19862.r30001

**Published:** 2019-05-30

**Authors:** Felix Silwimba

**Affiliations:** 1 University of Lusaka

This review has been migrated. The reviewer awarded 5 stars out of 5 This a relevant study particularly for accreditors in low middle-income countries. In this country mention of a visit by the accreditation agency inspectors is not a very welcome idea. However, as clearly mentioned in the study use of trained survey teams and volunteer peer reviewers is acceptable. Unlike the situation were survey teams are no nonsense uncompromising enforcers of the law. I have learnt a lot from this report.

Reviewer Expertise:

NA

No decision status is available
